# First person – Daniyal Jafree

**DOI:** 10.1242/dmm.052387

**Published:** 2025-04-28

## Abstract

First Person is a series of interviews with the first authors of a selection of papers published in Disease Models & Mechanisms, helping researchers promote themselves alongside their papers. Daniyal Jafree is first author on ‘ Microvascular aberrations found in human polycystic kidneys are an early feature in a *Pkd1* mutant mouse model’, published in DMM. Daniyal is a postdoctoral clinical research fellow in the lab of Professor David Long at UCL Great Ormond Street Institute of Child Health, London, UK, investigating the formation and function of the vasculature during development, health and disease.


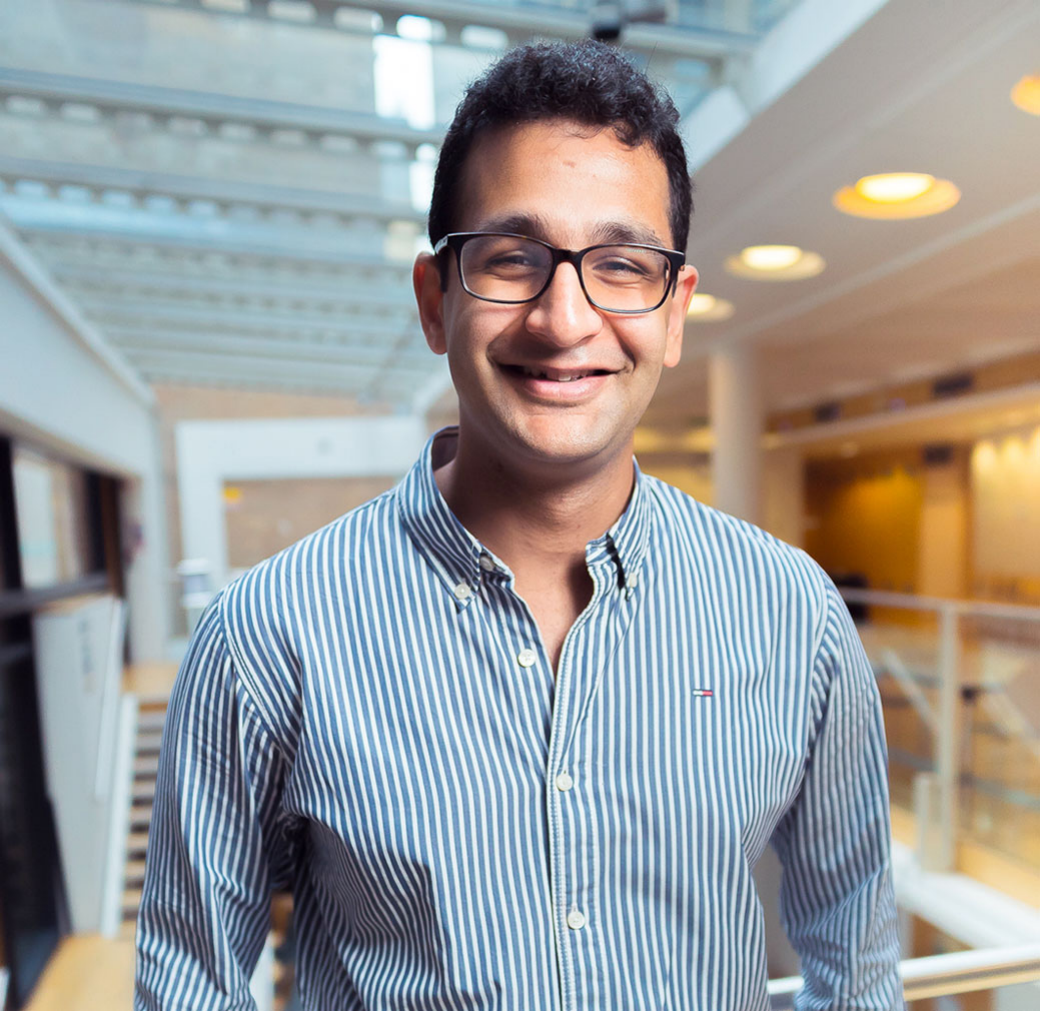


**Daniyal Jafree**

**Who or what inspired you to become a scientist?**

To be honest, I didn't always know that I would want to be a scientist. During medical school, I realised that my favourite part of the course was understanding the mechanisms behind human disease, and how these mechanisms lead to clinical symptoms and ultimately open doors to new treatments. The more I studied, the more I noticed huge gaps in what we know about disease biology. That realisation both perplexed me and excited me. I began exploring this further through clinical research, then summer lab internships, followed by a BSc and finally a PhD. I did eventually finish medical school (about 10 years after I started!).

Being a doctor can be incredibly fulfilling, but, during my PhD, I figured out that I also wanted to be the person uncovering why the disease happens in the first place, not just treating it. That sense of curiosity and purpose is what inspires me to keep going as a clinician–scientist today.

**What is the main question or challenge in disease biology you are addressing in this paper? How did you go about investigating your question or challenge?**

Autosomal dominant polycystic kidney disease (ADPKD) is the most common inherited kidney disease leading to dialysis or transplantation. While research has historically focused on the epithelial cysts characteristic of this condition, it's becoming clear that other non-epithelial cell types may play key roles in disease progression. For example, individuals with ADPKD are prone to systemic vascular abnormalities, such as cerebral aneurysms. However, how the specialised microvasculature within the kidney is affected, particularly from earlier stages of the disease, is not well known.

We hypothesised that kidney microvascular dysfunction might be more than just a late-stage phenomenon secondary to cyst expansion, but rather an early and intrinsic feature of ADPKD. To test this, we combined single-cell RNA sequencing, three-dimensional imaging with geometric and topological analyses, and functional magnetic resonance imaging (MRI) to study the kidney vasculature at molecular, structural and functional levels. We applied this to human ADPKD kidneys with advanced disease, and to a *Pkd1* mutant mouse model that allowed us to capture earlier disease stages.

In human ADPKD kidneys, we identified a distinct endothelial subpopulation with impaired angiogenic signalling and metabolic dysfunction. In the mouse model, similar endothelial changes, disorganised cortical microvasculature and reduced cortical perfusion were detectable early in disease, before loss of excretory function. Our findings suggest that kidney microvascular abnormalities arise early in ADPKD and represent a potential therapeutic target at stages when kidney function may still be preserved.

**How would you explain the main findings of your paper to non-scientific family and friends?**

Polycystic kidney disease (PKD) is a common genetic condition where fluid-filled cysts grow in the kidneys and eventually cause them to stop working, often requiring dialysis or a kidney transplant. Most research on PKD has focused on these cysts, but we wanted to know what else might be going wrong inside the kidney.

People with PKD often have blood vessel problems in other parts of the body, but it wasn't clear whether the tiny blood vessels inside the kidney were also affected. We thought these blood vessels, which are important for kidney function, might be in trouble early on, long before the kidneys fail.

To find out, we studied human kidneys with advanced PKD and used a mouse model that develops the same condition. We looked very closely at the kidney's blood vessels, specifically, their nature, their structure and how blood flows through them. In PKD, we found clear signs that the blood vessels inside the kidneys become damaged early and don't work properly, long before the kidneys shut down.

Due to these results, we think that these ‘messed up’ kidney blood vessels may play an important role in making PKD worse over time. This means that, in the future, we might be able to develop treatments that protect the blood vessels and help slow down the disease.

We found that microvascular abnormalities emerge early in disease, before noticeable decline in kidney function, suggesting a window for earlier therapeutic intervention.

**What are the potential implications of these results for disease biology and the possible impact on patients?**

Several important findings from previous studies set the stage for our work. For example, deletion of *Pkd1*, the gene most commonly mutated in ADPKD, disrupts vascular development and endothelial patterning. Other studies have shown abnormal microvascular architecture around cysts in both human ADPKD kidneys and rodent models, but mainly at late stages of disease. Clinical studies have also demonstrated that reduced kidney blood flow is linked to worsening kidney function in ADPKD.

However, key gaps remained: we didn't know the specific molecular changes occurring within the kidney vasculature, whether these were unique to ADPKD, or how early they appeared. Our study addressed these questions by analysing the ADPKD kidney vasculature at molecular, structural and functional levels using cutting-edge techniques.

We found that microvascular abnormalities emerge early in disease, before noticeable decline in kidney function, suggesting a window for earlier therapeutic intervention. Clinically, this potentiates targeting the vasculature to slow disease progression, for example, by restoring vascular health or improving blood flow. Our group has previously shown that delivering VEGF-C in a mouse model of PKD slows cyst growth, supporting the idea that the vasculature is a viable therapeutic target.

Our vision is to combine vascular-directed therapies with existing treatments, such as Tolvaptan, potentially offering a complementary strategy to preserve kidney function. The ultimate goal is to delay dialysis or the need for transplantation and improve long-term outcomes for patients with ADPKD.

**Figure:**
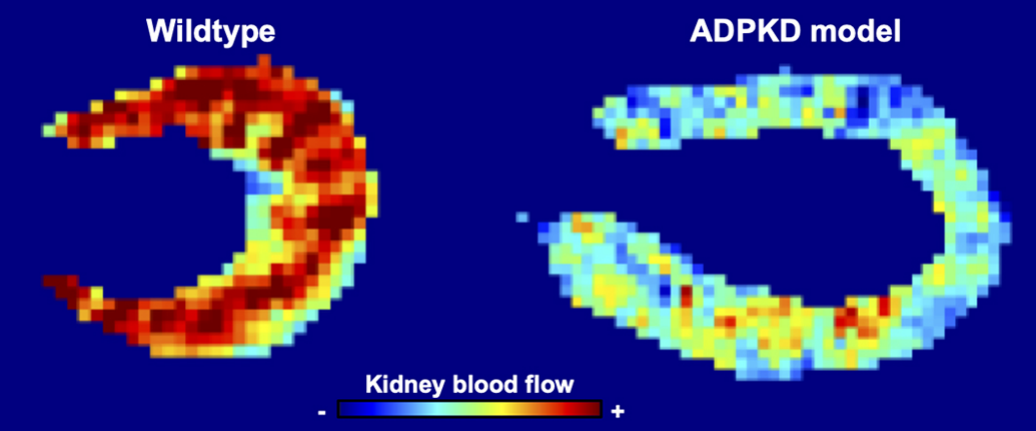
**Reduction of kidney blood flow is an early feature of polycystic kidney disease.** On the left is a kidney from a 3-month-old wild-type mouse, imaged using arterial spin labelling, an MRI-based technique that detects and quantifies blood flow. The velocity of blood flow is shown using a colour map, where red is the fastest and blue is the slowest. On the right is a kidney from a *Pkd1* mutant littermate. You can see the dramatic reduction in blood flow – occurring ∼6 months before we begin to see decline in excretory kidney function in this model. For me, this was the most striking of our experimental results. Raw images generated by Dr Charith Perera and Dr Daniele Tolomeo, UCL Centre for Advanced Biomedical Imaging.

**Why did you choose DMM for your paper?**

We chose DMM because of its remit for high-quality, translational research in disease biology. Our study spans molecular insights and advanced imaging of kidney microvascular structure and function, incorporating both human tissue and a clinically relevant animal model. We believe this aligns with DMM's commitment to publishing rigorous studies that integrate multiple types of data.

Additionally, we hope the DMM readership, including clinicians and scientists working on developmental biology, disease modelling and translational medicine in ADPKD research, would be interested in our findings. Our work introduces a new framework for analysing the kidney microvasculature and identifies early vascular abnormalities in ADPKD that may have clinical implications, so we were keen for it to be accessible to an audience interested in these translational aspects. DMM provides a supportive platform to communicate findings that bridge basic science and potential clinical impact.

On a side note, the editorial and review process at DMM was very constructive, helpful and fair!

**Given your current role, what challenges do you face and what changes could improve the professional lives of other scientists in this role?**

Balancing clinical work and research can be really challenging. There were weeks last year when I was working long shifts on busy hospital wards while also facing journal revisions and grant deadlines in the same week. Unfortunately, this is a scenario that many research-inclined clinicians are all too familiar with.

In the UK, there are dedicated clinical–academic training pathways that offer protected time for research, but these posts are extremely competitive and often limited to specific regions or research themes. As a result, you sometimes find yourself hoping the right job comes up at the right time, in the right place. There's growing concern about the declining clinical academic workforce in the UK, and I think more needs to be done to incentivise clinicians to get involved in research and to stay in it long term.

Until then, my best advice to aspiring clinical academics is to find a great mentor, a supportive lab environment, and a collaborative network. You'll notice our paper has many authors, each contributing unique expertise that helped push the project forward. Having a supervisor who encouraged me, gave constructive feedback, understood the realities of clinical work and allowed intellectual ‘breathing space’ made all the difference. It's this combination of mentorship, teamwork and understanding that has helped me continue pursuing translational research alongside clinical training.

There's growing concern about the declining clinical academic workforce in the UK […] more needs to be done to incentivise clinicians to get involved in research and to stay in it long term.

**What's next for you?**

I am both incredibly lucky and excited to have been awarded one of the new Wellcome Trust Accelerator Awards. It addresses some of the challenges I mentioned earlier by providing dedicated postdoctoral time to integrate my research into a clinical–academic career pathway.

In the short term, I'm completing the second year of my academic-themed resident doctor training in Cambridge, UK. During this time, I've had the opportunity to collaborate with scientists and clinicians at the Wellcome Sanger Institute and the University of Cambridge, working in new areas such as spatial transcriptomics, and analysing these complex datasets using generative AI models. Next year, I'll continue my research through the Accelerator Award while advancing my clinical training at Great Ormond Street Hospital for Children, an incredible environment to gain experience in paediatric medicine.

Longer term, my goal is to establish an independent research group focused on understanding and targeting the vasculature in disease. I'm particularly interested in developing translational approaches that bridge basic vascular biology with clinical application. I hope to build on my experiences, and the collaborative networks I've started to develop, to establish a research programme that uses computational approaches to inform and drive biological hypotheses. Ultimately, I'm aiming to answer fundamental questions in clinical medicine and human disease, with a focus on delivering meaningful benefits for patients.

**Tell us something interesting about yourself that wouldn't be on your CV**

One of my favourite hobbies is squash (the sport, not the vegetable!). I was introduced to it when I was about 10 years old and have loved both playing and following the professional game ever since.

At high school, I started our first squash team, often having to convince the rugby and hockey players to make up numbers when we didn't have enough for a match. Later, I helped run my medical school's squash team, and we were lucky enough to win the national medical school championships one year. More recently, I've joined Cambridge Squash Club and have been competing for their teams.

I'm particularly excited that squash has (finally) been introduced to the Olympics and will feature at the 2028 Games in Los Angeles. Leading up to that, I'm keen to give back to the sport, whether through charity initiatives, coaching or encouraging new players to get involved.

It's been a constant in my life outside of medicine and research and has helped me keep balance along the way. However, my life's great ambition is to get my partner into it too…but that's still a work in progress!

## References

[DMM052387C1] Jafree, D. J., Perera, C., Ball, M., Tolomeo, D., Pomeranz, G., Wilson, L., Davis, B., Mason, W. J., Funk, E. M., Kolatsi-Joannou, M. et al. (2025). Microvascular aberrations found in human polycystic kidneys are an early feature in a *Pkd1* mutant mouse model. *Dis. Model. Mech.* 18, dmm052024. 10.1242/dmm.052024 40114603 PMC12067086

